# Nanoarchitectonics of the cathode to improve the reversibility of Li–O_2_ batteries

**DOI:** 10.3762/bjnano.13.61

**Published:** 2022-07-21

**Authors:** Hien Thi Thu Pham, Jonghyeok Yun, So Yeun Kim, Sang A Han, Jung Ho Kim, Jong-Won Lee, Min-Sik Park

**Affiliations:** 1 Department of Advanced Materials Engineering for Information and Electronics, Integrated Education Institute for Frontier Science & Technology (BK21 Four), Kyung Hee University, 1732 Deogyeong-daero, Giheung-gu, Yongin 17104, Republic of Korea; 2 Department of Energy Science and Engineering, Daegu Gyeongbuk Institute of Science and Technology (DGIST), 333 Techno Jungang-daero, Hyeonpung-eup, Dalseong-gun, Daegu 42988, Republic of Korea; 3 Institute for Superconducting and Electronic Materials, Australian Institute for Innovative Materials, University of Wollongong, Squires Way, North Wollongong, NSW 2500, Australia

**Keywords:** cathode composition, electrochemistry, Li–O_2_ battery, metal–organic framework, nanoarchitectonics, zeolitic imidazolate framework

## Abstract

The strategic design of the cathode is a critical feature for high-performance and long-lasting reversibility of an energy storage system. In particular, the round-trip efficiency and cycling performance of nonaqueous lithium–oxygen batteries are governed by minimizing the discharge products, such as Li_2_O and Li_2_O_2_. Recently, a metal–organic framework has been directly pyrolyzed into a carbon frame with controllable pore volume and size. Furthermore, selective metallic catalysts can also be obtained by adjusting metal ions for outstanding electrochemical reactions. In this study, various bimetallic zeolitic imidazolate framework (ZIF)-derived carbons were designed by varying the ratio of Zn to Co ions. Moreover, carbon nanotubes (CNTs) are added to improve the electrical conductivity further, ultimately leading to better electrochemical stability in the cathode. As a result, the optimized bimetallic ZIF–carbon/CNT composite exhibits a high discharge capacity of 16,000 mAh·g^−1^, with a stable cycling performance of up to 137 cycles. This feature is also beneficial for lowering the overpotential of the cathode during cycling, even at the high current density of 2,000 mA·g^−1^.

## Introduction

Recently, lithium–oxygen batteries (LOBs) have received great attention as a future energy storage solution since they offer a tremendously high energy density compared to commercial lithium-ion batteries (LIBs) [1–2]. An aprotic LOB is composed of a porous air cathode and a metallic Li anode, which are separated by a porous separator containing a Li^+^-conductive aprotic electrolyte. In principle, electrochemical reactions between Li^+^ and O_2_ take place in the cathode to store and convert energy. During the discharge, the oxygen reduction reaction (ORR) occurs at the surface of the cathode, where O_2_ is spontaneously reduced by Li^+^ coming from the metallic Li anode, leading to the formation of Li_2_O_2_ as the final discharge product. During the subsequent charge, Li_2_O_2_ can be reversibly decomposed to Li^+^ and O_2_ by the oxygen evolution reaction (OER) [3–6]. Considering these reaction mechanisms of LOBs, the cathode should have a large surface area to offer abundant active sites for the electrochemical reactions and a large pore volume for effective accommodation of Li_2_O_2_ [7–8]. If accumulated Li_2_O_2_ is not completely decomposed during the charge, the reaction sites and diffusion pathways of electrolytes and oxygen species are blocked, resulting in a decrease of the electrochemical performance of LOBs [9]. In addition to the pore structure of the cathode, the morphology and physicochemical properties of Li_2_O_2_ directly affect the overpotential and round-trip efficiency of LOBs during cycling [10–11]. The morphologies and physicochemical properties of Li_2_O_2_ are reported to be dependent on many factors, including the pore structure of cathode. The electrical conductivity of the cathode is also essential for securing long-term cyclability as well as rate capability [12]. In this respect, various types of carbon materials have been explored as advanced cathode materials for LOBs, owing to their controllable pore structure with a high surface-to-volume ratio and excellent electrical conductivity [13–14]. In particular, nitrogen-doped carbon materials have shown electrocatalytic activity towards the ORR and/or OER, which would be a favorable characteristic for the effective reduction of the overpotential at the cathode of LOBs [15–16].

Recently, metal–organic framework (MOF)-derived carbon materials have received great attention as potential candidates due to their controllable compositions and porous structures [17–29]. In particular, zeolitic imidazolate frameworks (ZIFs), in which metal ions are coordinated by 2-methylimidazolate linkers, have shown some initial promise in terms of porosity and chemical stability [30–34]. In practice, ZIF-8 and ZIF-67 (in which the metal ions are zinc and cobalt, respectively) have been extensively studied for various energy storage applications [35–36]. From a structural viewpoint, ZIF-8-derived carbon materials have a large specific surface area with a well-defined microporous structure and a high N content [37]. Meanwhile, ZIF-67-derived carbon materials have a higher mesopore volume and higher degree of graphitization, showing excellent electrical conductivity. Moreover, the inevitable Co residue in the structure would be helpful for facilitating the electrochemical reactions by lowering the overpotential during cycling [38]. Herein, we demonstrate bimetallic ZIF-derived carbon materials to combine the advantages of both ZIF-8- and ZIF-67-derived carbon materials. For practical use, bimetallic ZIFs with different ratios of Zn/Co were directly grown on carbon nanotubes (CNTs) to secure electrical conductivity and sufficient diffusion pathways for oxygen and electrolyte in the cathode. The ratio of Zn/Co in the starting materials greatly affects the microstructure and porosity of the resulting bimetallic ZIF–carbon/CNT composites. The correlation between the microstructure and the electrochemical performance of the bimetallic ZIF–carbon/CNT composites has been thoroughly investigated for their practical use as potential cathode materials for high-performance LOBs.

## Results and Discussion

**Figure 1:**
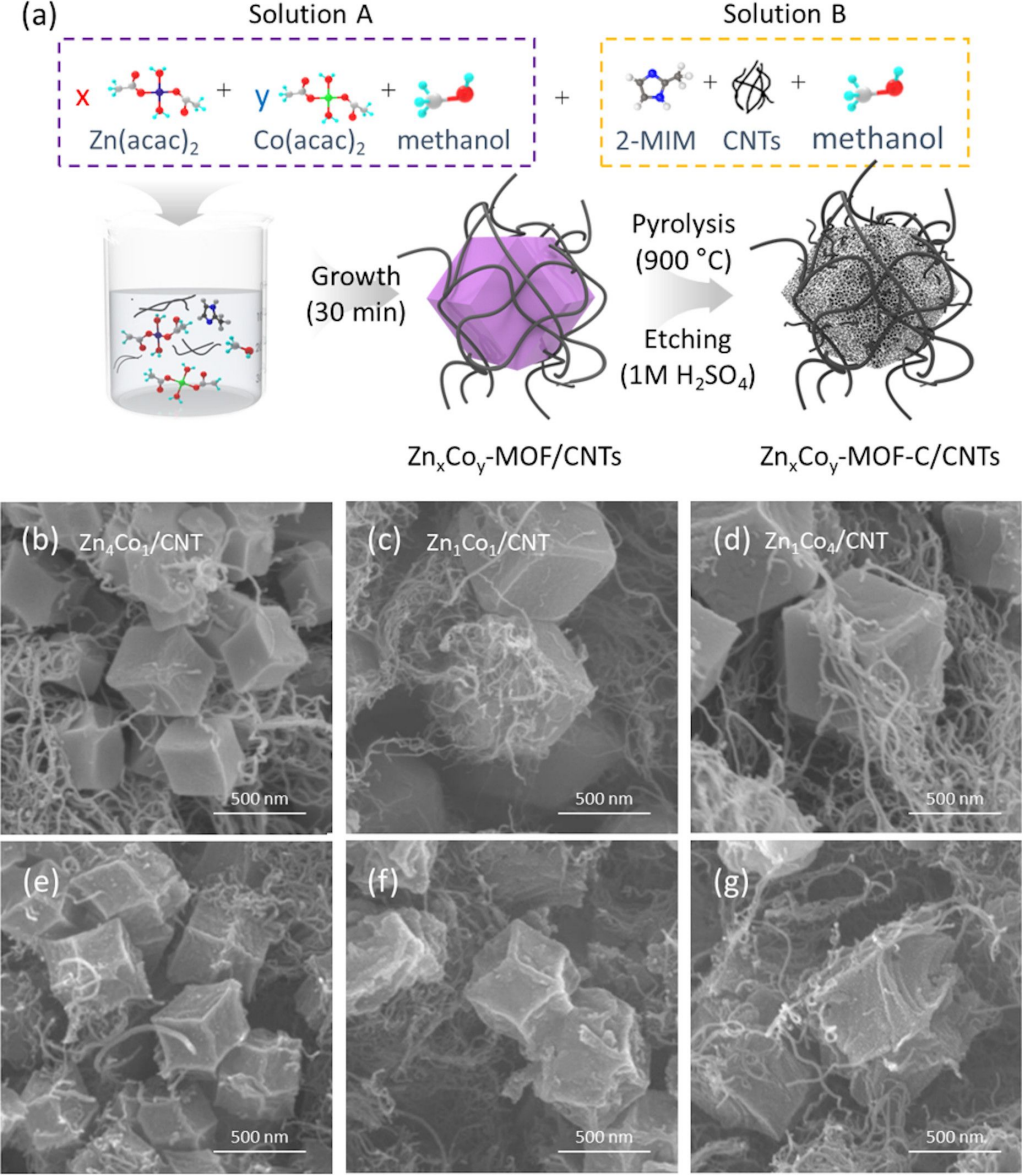
(a) Design and synthesis of Zn*_x_*Co*_y_*–CNT. FESEM images of (b, c, d) as-prepared and (e, f, g) etched composites. (b, e) Zn_4_Co_1_–CNT, (c, f) Zn_1_Co_1_–CNT, and (d, g) Zn_1_Co_4_–CNT.

In pursuit of improving the reversibility of LOBs, a bimetallic ZIF (Zn*_x_*Co*_y_*) was designed and grown on CNTs via hydrothermal synthesis using Zn and Co acetates, together with 2-methylimidazolate, as described in Figure 1a. The hydrothermal process is beneficial for facilitating the nucleation and growth of bimetallic ZIF as well as reducing the synthesis time. The chemical composition of Zn*_x_*Co*_y_* particles was controlled by adjusting the ratio of Zn/Co (*x*/*y* = 1/4, 1/1, and 4/1) in the starting materials. After carbonization at 900 °C and chemical etching with 1 M H_2_SO_4_, bimetallic Zn*_x_*Co*_y_*–C/CNT composites were successfully obtained to be used as cathode materials for LOBs.

Figure 1b–d shows the morphologies of bimetallic Zn_4_Co_1_, Zn_1_Co_1_, and Zn_1_Co_4_ particles grown on CNT frameworks, respectively. Field-emission scanning electron microscopy (FESEM) observations confirmed that abundant rhombic dodecahedral Zn*_x_*Co*_y_* particles with different Zn/Co ratios were successfully integrated into the CNTs. Corresponding energy-dispersive X-ray spectroscopy (EDS) elemental mapping results (Supporting Information File 1, Figure S1) confirm that Zn and Co were uniformly distributed inside the as-grown Zn*_x_*Co*_y_* particles. We also found that the size of the Zn*_x_*Co*_y_* particles was decreased by increasing the ratio of Zn/Co during the synthesis due to the distinctive formation mechanisms of the parental ZIF-8 and ZIF-67 particles. Under the same synthesis conditions, the particle size of ZIF-8 is always smaller than that of ZIF-67. This is because the formation of ZIF-67 is proceeded by a fast one-step growth mechanism while ZIF-8 is formed by a slower two-step growth mechanism (i.e., nucleation and growth) [39]. The different formation mechanisms are mainly responsible for determining the particle sizes of ZIF-8 and ZIF-67.

After carbonization and chemical etching processes, we obtained a series of Zn*_x_*Co*_y_*–C/CNT composites, as shown in Figure 1e–1g, in which the highly porous Zn*_x_*Co*_y_*–C particles are beneficial for facilitating the electrochemical reactions between Li^+^ and O_2_. Moreover, CNT networks allow for sufficient electronic conduction as well as diffusion pathways for O_2_ and electrolyte in the composites. The atomic ratios of Zn and in the Zn_1_Co_4_–C/CNT, Zn_1_Co_1_–C/CNT, and Zn_4_Co_1_–C/CNT composites were measured by EDS elemental mapping (Supporting Information File 1, Figure S2). Moreover, it should be noted that all the Zn*_x_*Co*_y_*–C particles exhibited a high concentration of N, mainly induced by thermal decomposition of 2-methylimidazole during the carbonization process. After the carbonization and chemical etching processes, the sizes of the Zn*_x_*Co*_y_* particles were slightly decreased due to the thermal evaporation of organic linkers and metal ions, maintaining free spaces in the particles.

**Figure 2:**
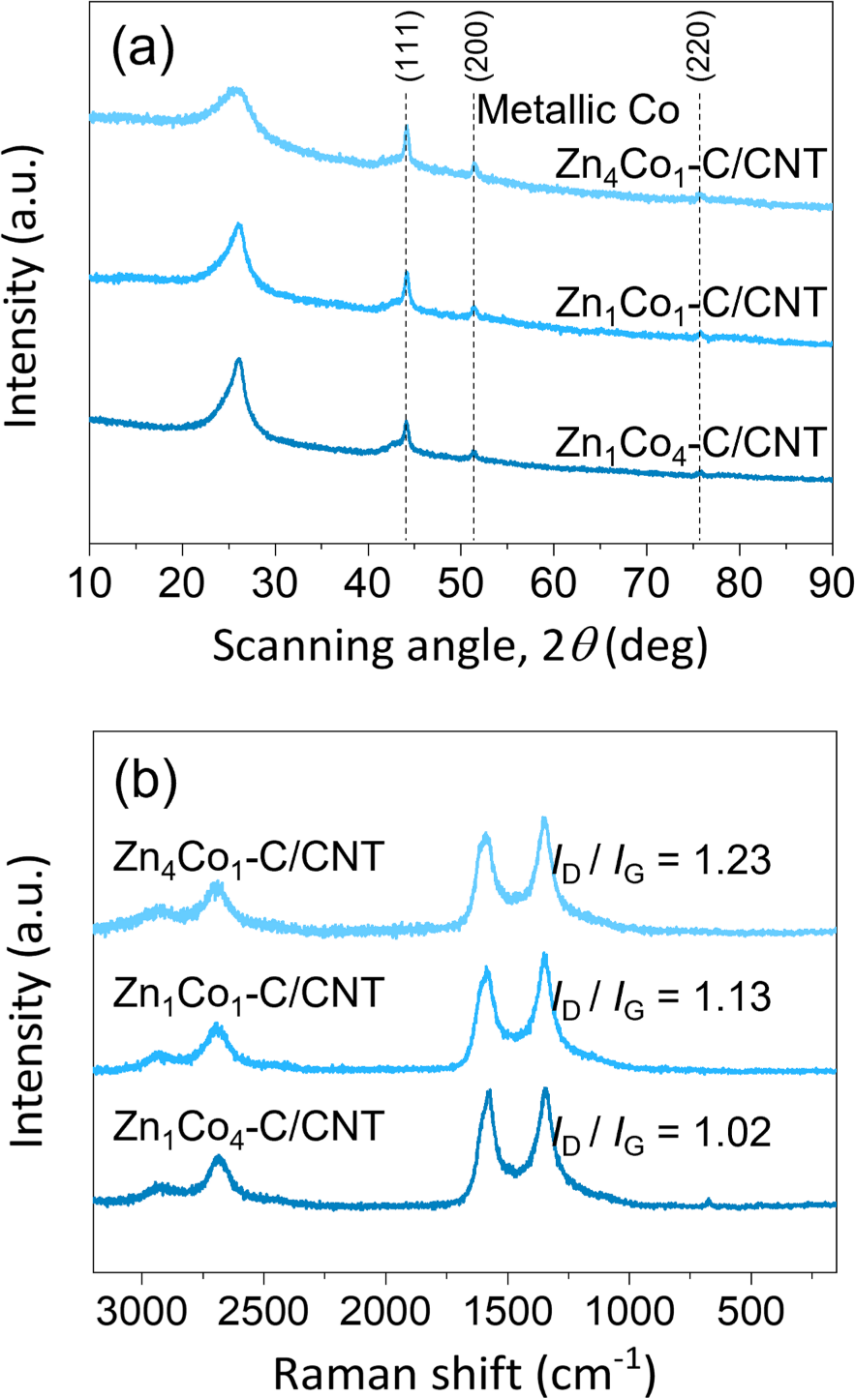
(a) XRD patterns and (b) Raman spectra of Zn*_x_*Co*_y_*–C/CNT composites.

According to the X-ray diffraction (XRD) patterns of the as-grown Zn*_x_*Co*_y_* particles on the CNT framework, all reflections are well matched with those of simulated patterns of ZIF-8 and ZIF-67 (Supporting Information File 1, Figure S3). After carbonization and chemical etching, the XRD patterns of Zn*_x_*Co*_y_*–C/CNT composites exhibit a strong signal for the (002) reflection of graphitic carbon at around 26° with a trace of metallic Co (Figure 2a). Multiple characteristic peaks are detected around 44.1°, 51.4°, and 75.7°, which correspond to (111), (200), and (220) reflections of metallic Co, respectively. Moreover, we found that the (002) peak became obviously sharper in the composites with a decreasing Zn/Co ratio during synthesis. This is because Co facilitates the graphitization of Zn*_x_*Co*_y_* particles during the carbonization process. From the results, we confirm the critical role of Co for tailoring the microstructure of Zn*_x_*Co*_y_*–C particles in the composites.

Figure 2b compares Raman spectra of Zn*_x_*Co*_y_*–C/CNT composites, showing typical Raman bands at ≈1346 cm^−1^ (D band), ≈1576 cm^−1^ (G band), and ≈2680 cm^−1^ (2D band). All the composites show similar Raman scattering without a noticeable difference in full width at half maximum (FWHM) values. Assuming the same content of CNTs in the composites, the differences in the intensity ratio of the D to the G band (*I*_d_/*I*_g_) are mainly attributable to the crystallinity of the Zn*_x_*Co*_y_*–C particles. The Zn_1_Co_4_–C/CNT composite has the best crystallinity with a ratio of 1.02, which is lower than that of the Zn_1_Co_1_–C/CNT (1.13) and Zn_4_Co_1_–C/CNT (1.23) composites.

**Figure 3:**
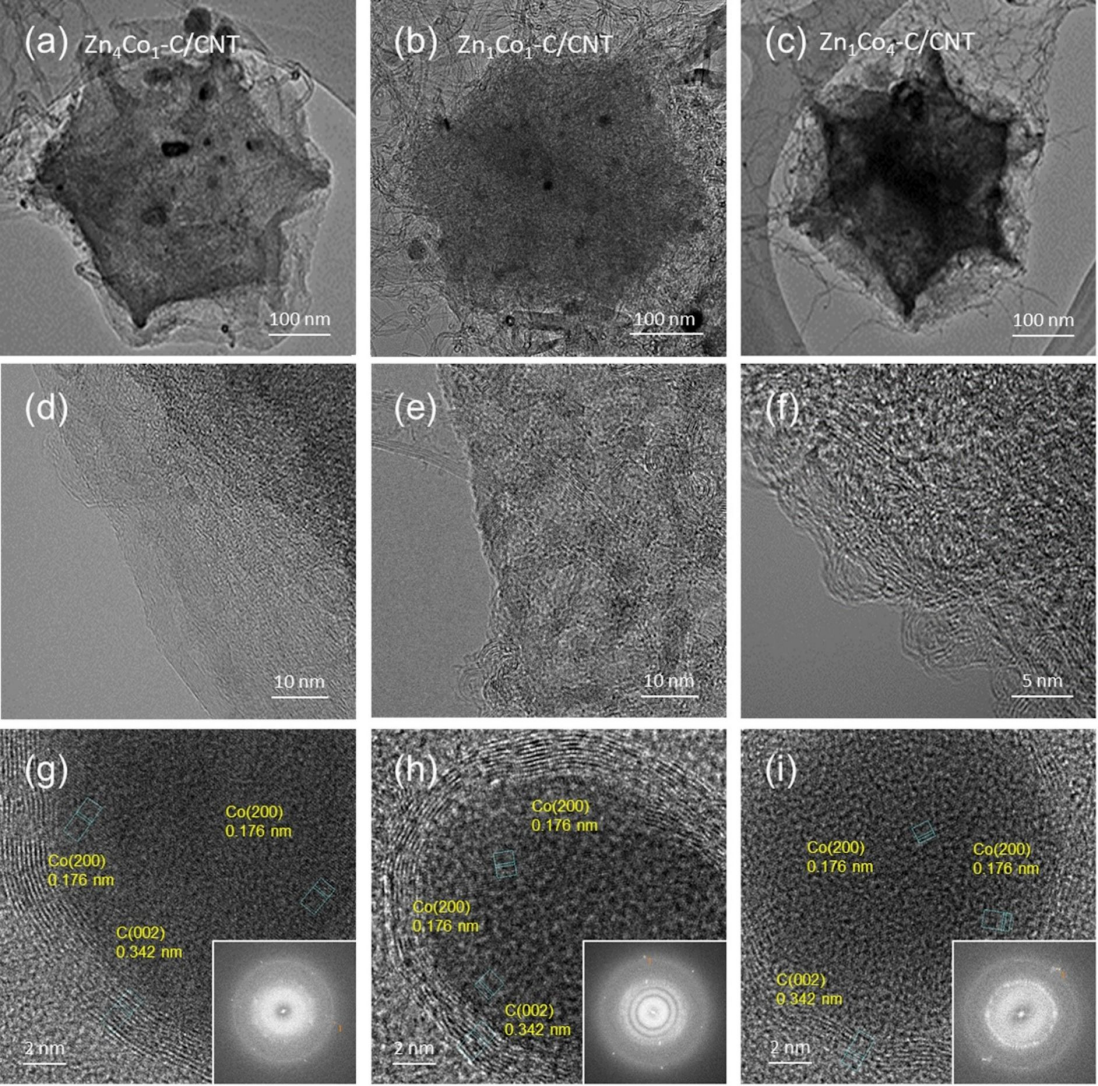
TEM images of (a, d, g) Zn_4_Co_1_–C/CNT, (b, e, h) Zn_1_Co_1_–C/CNT, (c, f, i) Zn_1_Co_4_–C/CNT. The insets in the bottom row are the corresponding selected area diffraction (SAED) patterns.

Transmission electron microscopy (TEM) observations with corresponding fast Fourier transform (FFT) patterns confirm the different crystallinity of Zn*_x_*Co*_y_*–C particles, depending on the concentration of Co during the synthesis. After the carbonization, the crystallinity of Zn*_x_*Co*_y_*–C particles can be enhanced by decreasing the Zn/Co ratio during synthesis [35]. Unlike Zn_4_Co_1_–C particles (Figure 3a), which have a typical amorphous carbon structure, both Zn_1_Co_1_–C (Figure 3b) and Zn_1_Co_4_–C (Figure 3c) particles contain some short-range graphitic carbon structures with a lattice (*d*)-spacing of 0.34 nm. Even after chemical etching, we still found residual metallic Co in the Zn*_x_*Co*_y_*–C/CNT composites with a *d*-spacing of 0.176 nm, corresponding to (200) crystal planes of the face-centered cubic structure (Figure 3d–f). The amount of residual metallic Co is dependent on the chemical composition of Zn*_x_*Co*_y_* particles. It is expected that it would play an important role as an electrocatalyst in the composites for lowering the overpotential for the given electrochemical reactions.

**Figure 4:**
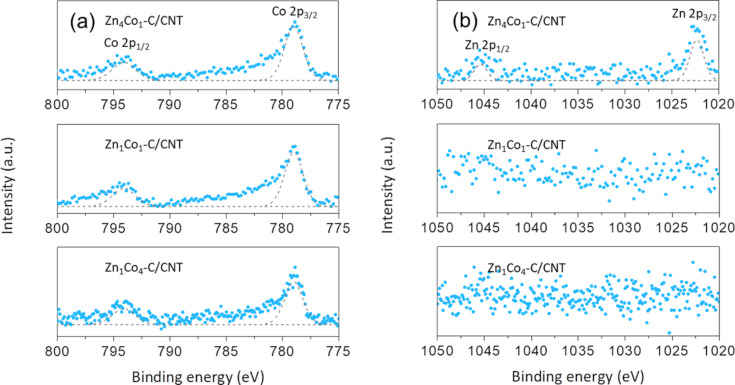
XPS spectra of Zn*_x_*Co*_y_*–C/CNT composites: (a) Co 2p and (b) Zn 2p.

For further inspection of the structural characteristics, the surface chemistry of Zn*_x_*Co*_y_*–C/CNT composites was further investigated by using X-ray photoelectron spectroscopy (XPS), as shown in Figure 4. The XPS spectra were carefully deconvoluted, based on the excitation of C 1s at the binding energy of 284.5 eV. According to the Co 2p spectra collected from the Zn*_x_*Co*_y_*–C/CNT composites (Figure 4a), strong signals were observed at binding energies of 778.9 and 794 eV, corresponding to the Co 2p_3/2_ and Co 2p_1/2_ orbitals of metallic Co, respectively, regardless of the Zn/Co ratio. In contrast, the signal of Zn was only detected in the Zn 2p spectrum of the Zn_4_Co_1_–C/CNT composite (Figure 4b), indicating that Zn was easily evaporated during the carbonization process. From the C 1s and N 1s spectra (Supporting Information File 1, Figure S4), we found that N species could be spontaneously doped by the thermal decomposition of 2-methylimidazolate during carbonization. In practice, Zn_1_Co_4_/CNT composites show the highest sp^2^-carbon content induced by the highest crystallinity among the Zn*_x_*Co*_y_*–C particles, in which the N content was measured to be 2.3 atom %. It is expected that such structural features would promote high electrical conductivity of the Zn*_x_*Co*_y_*–C particles. Therefore, it can be inferred that the crystallinity and N content in the Zn*_x_*Co*_y_*–C particles can be increased by decreasing the Zn/Co ratio, since the metallic Co facilitates graphitization and N doping at a given temperature.

**Figure 5:**
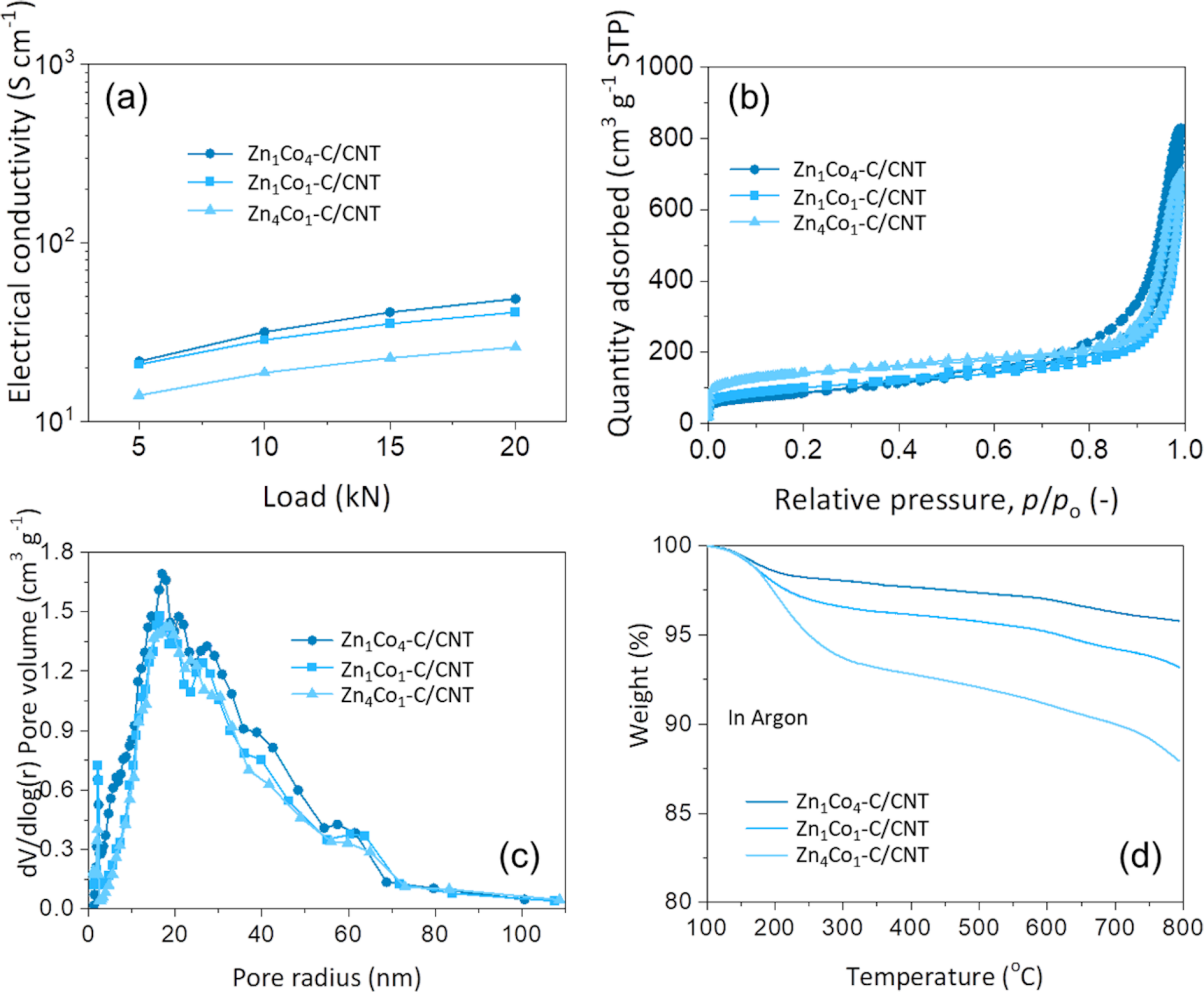
(a) Electrical conductivity, (b) N_2_ sorption isotherms, (c) Dollimore–Heal desorption pore size distributions, and (d) TGA curves of Zn_x_Co_y_–C/CNT.

Figure 5a shows the electrical conductivities of Zn*_x_*Co*_y_*–C/CNT composites, indicating that the Zn_1_Co_4_–C/CNT composite with the smallest Zn/Co ratio showed a superior electrical conductivity of 48.5 S·cm^−1^. This is attributed to the highest crystallinity of the Zn_1_Co_4_–C/CNT composite, which contains the highest fraction of metallic Co among the given structures. The pore structures of the Zn*_x_*Co*_y_*–C/CNT composites were characterized by N_2_ isotherms, as presented in Figure 5b. The specific surface areas and total pore volumes of Zn*_x_*Co*_y_*–C/CNT composites were calculated by using the Brunauer–Emmett–Teller (BET) model. Note that the pore structure of the Zn*_x_*Co*_y_*–C/CNT composites is highly dependent on the Zn/Co ratio during synthesis. The composite with higher Zn content has a larger BET surface area and micropore volume but a smaller mesopore volume, as compared in Figure 5c and Supporting Information File 1, Table S1. According to the adsorption–desorption hysteresis curves, the Zn_1_Co_4_–C/CNT composite has the lowest BET surface area (305 m^2^·g^−1^) but the highest mesopore volume (1.23 cm^3^·g^−1^). In contrast, the Zn_4_Co_1_–C/CNT composite shows the highest BET surface area (489 m^2^·g^−1^) with the lowest mesopore volume (0.88 cm^3^·g^−1^). This reveals that Zn*_x_*Co*_y_*–C particles possess typical characteristics of both ZIF-8- and ZIF-67-derived carbon materials. During the carbonization process, Zn forms a microporous domain with a large surface area while Co forms a mesoporous domain with a small surface area in the carbon matrix. Therefore, the surface area can be reduced with an increase in mesopore volume by increasing the Co concentration. Thus, we note that the BET surface area and mesopore volume of Zn*_x_*Co*_y_*–C/CNT composite can be easily tailored by controlling the Zn/Co ratio.

On the other hand, the thermal stability of Zn*_x_*Co*_y_*–C/CNT composites was also examined by thermogravimetric analysis (TGA), as presented in Figure 5d. From the TGA curves, the weight losses were measured to be 6.1%, 9.2%, and 17.4% for Zn_1_Co_4_–C/CNT, Zn_1_Co_1_–C/CNT, and Zn_4_Co_1_–C/CNT composites, respectively. These weight losses were mainly due to thermal evaporation of adsorbed moisture and Zn, together with thermal decomposition of amorphous carbon in the Zn*_x_*Co*_y_*–C particles. In this respect, the Zn_1_Co_4_–C/CNT composite was the most thermally stable because of its relatively higher fraction of robust graphitic carbon structure.

**Figure 6:**
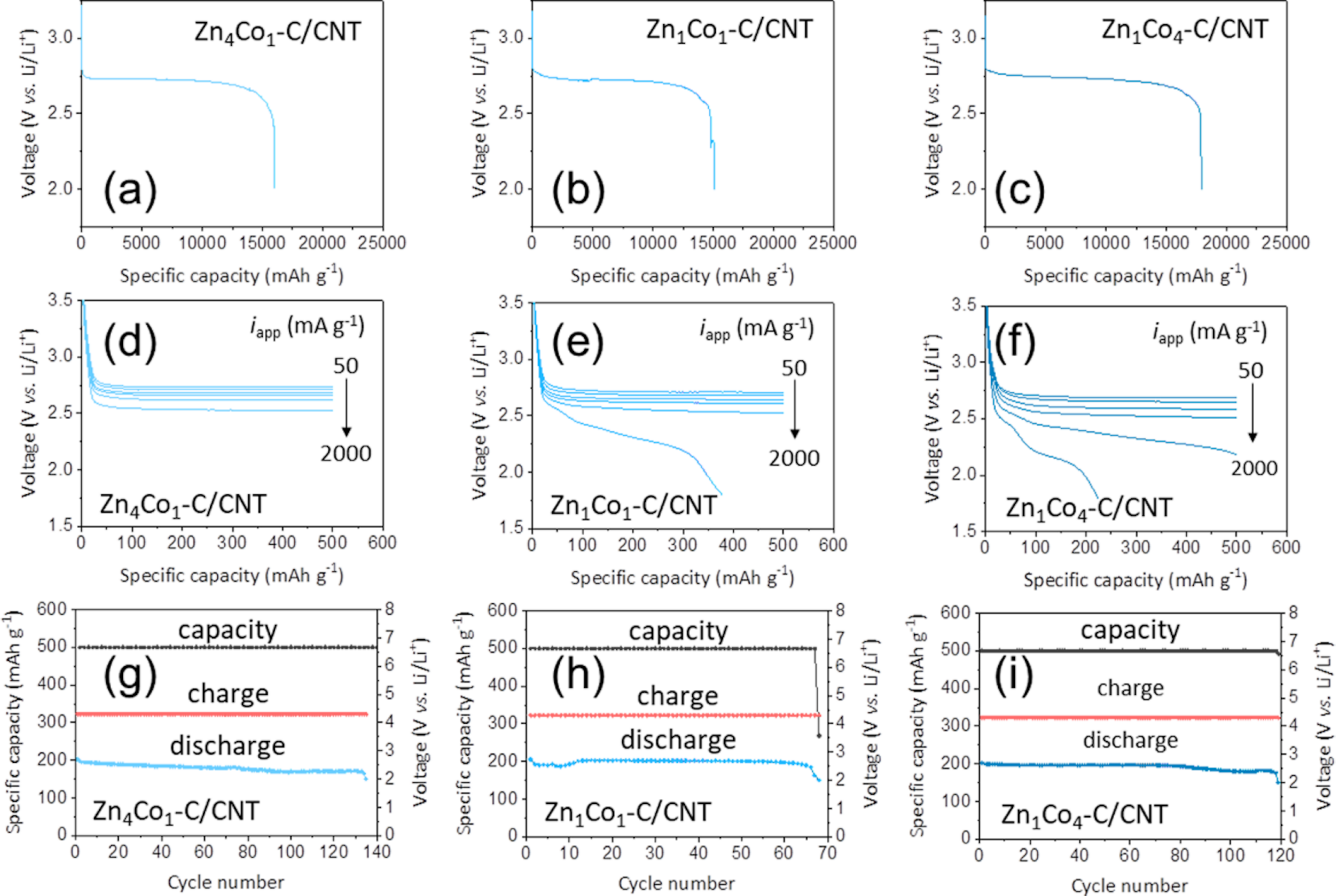
Galvanostatic discharge profiles (a, b, c) of the LOBs with (a) Zn_4_Co_1_–C/CNT, (b) Zn_1_Co_1_–C/CNT, and (c) Zn_1_Co_4_–C/CNT electrodes. The discharge measurements were performed in pure O_2_ at a current density of 50 mA·g^−1^. Plots of discharge curves (d, e, f) of (d) Zn_4_Co_1_–C/CNT, (e) Zn_1_Co_1_–C/CNT, and (f) Zn_1_Co_4_–C/CNT electrodes. The cells were discharged in pure O_2_ with a limited capacity of 500 mAh·g^−1^ at various current densities in the range of 50–2,000 mA·g^−1^. The terminal discharge and charge potentials during cycling (g, h, i) measured on (g) Zn_4_Co_1_–C/CNT, (h) Zn_1_Co_1_–C/CNT, and (i) Zn_1_Co_4_–C/CNT electrodes. The measurements were performed in pure O_2_ at a current density of 200 mA·g^–1^ with a cutoff capacity of 500 mAh·g^−1^.

Figure 6a–c shows the galvanostatic discharge profiles of the LOBs assembled with Zn*_x_*Co*_y_*–C/CNT cathodes at a current density of 50 mA·g^−1^. All of the cathodes showed a well-defined voltage plateau at ≈2.7 V vs Li/Li^+^ for Li_2_O_2_ formation. The initial discharge capacities of Zn_4_Co_1_–C/CNT, Zn_1_Co_1_–C/CNT, and Zn_1_Co_4_–C/CNT cathodes were measured to be 16,000 mAh·g^−1^, ≈15,100 mAh·g^−1^, and ≈17,900 mAh·g^−1^, respectively. These results support the premise that the specific surface area and total pore volume of Zn*_x_*Co*_y_*–C/CNT directly affect the formation of Li_2_O_2_ in the cathodes. In particular, the highest initial discharge capacity of the Zn_1_Co_4_–C/CNT cathode is mainly attributable to the large total pore volume of (1.23 cm^3^·g^−1^) rather than to the specific surface area (305 m^2^·g^−1^). This is because Li_2_O_2_ is formed on the cathode surface at the initial state of discharge and then deposited in the pores during the discharge process.

The rate capability of Zn*_x_*Co*_y_*–C/CNT cathodes was also investigated, as shown in Figure 6d–f. The galvanostatic discharge profiles of Zn*_x_*Co*_y_*–C/CNT cathodes were recorded with a limited capacity of 500 mAh·g^−1^ at various current densities (*i*_app_) ranging from 50 to 2,000 mA·g^−1^. Interestingly, Zn*_x_*Co*_y_*–C/CNT cathodes exhibited different trends in their rate capability compared with the discharge capacity. With an increasing current density, the specific surface area of Zn*_x_*Co*_y_*–C/CNT cathodes becomes more crucial for boosting electrochemical reactions and lowering the overpotential for Li_2_O_2_ formation. In practice, the Zn_4_Co_1_–C/CNT cathode with the highest specific surface area maintained a stable electrochemical performance even at a high current density of 2,000 mA·g^−1^, while the Zn_1_Co_1_–C/CNT and Zn_1_Co_4_–C/CNT cathodes showed significant fading in their performance. According to plots of the overpotential (η) values for various cathodes against *i*_app_ (Supporting Information File 1, Figure S5), we note that the specific surface area of Zn*_x_*Co*_y_*–C/CNT is the predominant factor determining the rate capability of LOBs, because at high current densities, the oxygen cannot diffuse deeply into the pores and Li_2_O_2_ tends to form film-like particles on the surfaces of cathodes [40].

Even though the Zn_4_Co_1_–C/CNT cathode has the lowest electrical conductivity, it still exhibited the lowest η value. This implies that the overpotential is not simply dependent on the electrical conductivity of the cathode, but is also related to the nature of the discharge product, Li_2_O_2_, as well as to electrocatalytic effects. In the Zn_4_Co_1_–C/CNT cathode with a large specific surface area, the formation of amorphous Li_2_O_2_ film could be favored rather than that of toroid-like Li_2_O_2_. Compared with toroid-like Li_2_O_2_, amorphous Li_2_O_2_ film has better ionic conductivity; therefore, it is able to effectively reduce the charge transfer resistance. In addition, the electrocatalytic activities of metallic Co and N could facilitate the decomposition of Li_2_O_2_ during the next charge process, so that the overpotential of Zn_4_Co_1_–C/CNT cathode could be reduced.

The cycling performance of Zn*_x_*Co*_y_*–C/CNT cathodes was examined at a current density of 200 mA·g^−1^, as shown in Figure 6g–i. The Zn_4_Co_1_–C/CNT cathode exhibited superior cycling stability (≈137 cycles) compared to that of the Zn_1_Co_1_–C/CNT cathode (≈70 cycles) and Zn_1_Co_4_–C/CNT cathode (≈120 cycles). Even after 100 cycles, the Zn_4_Co_1_–C particles still maintained their morphologies and microstructures without significant structural deterioration (Supporting Information File 1, Figure S6). This could be attributed to the relatively low overpotential of the Zn_4_Co_1_–C/CNT cathode during cycling, which facilitates the reversible formation and decomposition of Li_2_O_2_. Thus, it should be emphasized that the pore structure control of cathode materials is the main responsible for improving the long-term cycling performance of LOBs (Supporting Information File 1, Table S2).

## Conclusion

In summary, bimetallic Zn*_x_*Co*_y_*–C/CNT composites with various Zn/Co ratios are proposed as promising cathode materials for LOBs. The surface area and pore volume of the Zn*_x_*Co*_y_*–C/CNT composites could be controlled by adjusting the Zn/Co ratio during synthesis. The chemical composition of Zn*_x_*Co*_y_* particles directly affects their physicochemical properties such as BET surface area, pore volume, crystallinity, and electrical conductivity. Such controllable structural characteristics of the Zn*_x_*Co*_y_*–C/CNT composites is beneficial for designing a robust cathode material to boost the electrochemical reactions as well as improving the reversibility of LOBs. Based on the compositional optimization of Zn*_x_*Co*_y_* particles, we demonstrated an extremely high discharge capacity of ≈16,000 mAh·g^−1^ for the Zn_4_Co_1_–C/CNT composite cathode and stable cycling performance up to 137 cycles at a current density of 200 mA·g^−1^. We believe that our findings could offer useful design principles for advanced cathode materials for high-performance LOBs.

## Experimental

### Material preparation

In a manner similar to our previous works [12,41], bimetallic ZIFs (Zn*_x_*Co*_y_*) with various Zn/Co ratios were grown on CNTs via hydrothermal synthesis, where *x*/*y* was chosen to be 1/4, 1/1, and 4/1, respectively (corresponding to 20%, 50%, and 80% of zinc ions in the metal precursors). Firstly, metal solutions were prepared by dissolving stoichiometric amounts of cobalt acetate tetrahydrate (Co(CH_3_COO)_2_⋅4H_2_O, Sigma-Aldrich, 98%) and zinc acetate dihydrate (Zn(CH_3_COO)_2_·2H_2_O, Sigma-Aldrich, 99%) in methanol (60 mL). Meanwhile, 59 mg of CNTs with diameters of 15–30 nm were dispersed in methanol (60 mL) containing 2.6 g of 2-methylimidazole (C_4_H_6_N_2_) by horn sonication for 30 min. After that, the metal solution was poured into the as-prepared CNT-containing solution. The mixture was continuously stirred for 30 min and then poured into a 200 mL Teflon-lined autoclave for heating at 90 °C for 6 h. After the temperature naturally dropped down to room temperature, the Zn*_x_*Co*_y_*/CNT precipitate was collected by vacuum filtration, washed with methanol, and then dried at 80 °C. After carbonization at 900 °C for 6 h under Ar atmosphere, the Zn*_x_*Co*_y_*–C/CNT composite was obtained and further chemically etched with 1 M of H_2_SO_4_ solution before use.

### Material characterization

Field-emission scanning electron microscopy (JEOL, JSM-7000F) and high-resolution TEM (HRTEM, JEOL, JEM-2100F) with EDS were used to examine the morphologies and microstructures of the materials. Powder XRD (PANalytical, Empyrean) and Raman spectroscopy (inVia Raman microscopes, Ar ion laser, 514 nm) were employed to analyze the structures. Their surface chemistry was investigated by XPS (Thermo Scientific, Sigma Probe), while their surface area and porosity were determined by a porosity analyzer (Micromeritics, Tristar II 3020). The electrical conductivity measurements were conducted by the four-point probe method using a power resistivity measurement system (MCP-PD51) at different applied pressures ranging from 5 to 20 kN. The thermal stability was examined by TGA (Thermal Analyzer, TGA Q5000 IR) with a scan rate of 5 °C·min^−1^ under Ar atmosphere.

### Electrochemical experiments

The electrochemical performance of cathode materials was investigated using a coin-type LOB cell composed of a Li metal anode, a liquid electrolyte impregnated into a glass-fiber separator, and a cathode. The cells were assembled in an Ar-filled glove box, and 1 M of lithium bis(trifluoromethanesulfonyl)imide (LiTFSI) dissolved in tetraethylene glycol dimethyl ether (TEGDME) was used as the liquid electrolyte. For the cathode preparation, the Zn*_x_*Co*_y_*–C/CNT composite was dispersed in deionized water by horn sonication for 1 h. The suspension was filtered through a glass-fiber membrane (pore diameter of 1.2 μm) without any polymeric binder and conductive agent. The active area and mass loading of the cathode were 0.785 cm^2^ and 1.0 mg·cm^−2^, respectively. Porous Ni foam was placed on the cathode side to enable uniform distribution of O_2_ gas as well as efficient current collection. The cell was assembled in a glove box filled with purified Ar gas. Then, it was placed in a gas-tight chamber with a controlled gas flow rate and pressure, and high-purity O_2_ gas (99.99%) was supplied to the chamber. Galvanostatic discharge profiles were measured at a current density of 50 mA·g^−1^ (based on the cathode mass) by means of a battery tester (WonATech, WBCS3000S). For rate-capability tests, the cell was discharged at various current densities in the range of 50–2000 mA·g^−1^. After discharge, the cell was charged using a constant current (CC)–constant voltage (CV) protocol (i.e., CC charge at 50 mA·g^−1^ to 4.3 V vs Li/Li^+^ followed by CV charge with a 5 mA·g^−1^ cutoff current and 500 mAh·g^−1^ cutoff capacity). For long-term cycling tests, the cell was discharged with a limited capacity of 500 mAh·g^−1^ at 200 mA·g^−1^, which was followed by a CC–CV charge (i.e., CC charge at 200 mA·g^−1^ to 4.3 V vs Li/Li^+^ and CV charge with a 20 mA·g^−1^, cutoff current, and 500 mAh·g^−1^, cut-off capacity, whichever occurred first). All electrochemical experiments were performed at 25 °C.

## Supporting Information

File 1 Additional TEM, EDS, XRD, XPS, BET, SEM, and cathodic overpotential measurements.

## References

[1] Grande L, Paillard E, Hassoun J, Park J-B, Lee Y-J, Sun Y-K, Passerini S, Scrosati B (2015). Adv Mater (Weinheim, Ger).

[2] Aurbach D, McCloskey B D, Nazar L F, Bruce P G (2016). Nat Energy.

[3] Lu Y-C, Gallant B M, Kwabi D G, Harding J R, Mitchell R R, Whittingham M S, Shao-Horn Y (2013). Energy Environ Sci.

[4] Padbury R, Zhang X (2011). J Power Sources.

[5] Geng D, Ding N, Hor T S A, Chien S W, Liu Z, Wuu D, Sun X, Zong Y (2016). Adv Energy Mater.

[6] Balaish M, Jung J-W, Kim I-D, Ein‐Eli Y (2020). Adv Funct Mater.

[7] Zhai D, Wang H-H, Yang J, Lau K C, Li K, Amine K, Curtiss L A (2013). J Am Chem Soc.

[8] Zhou Y, Zhao Y, Liu Z, Peng Z, Wang L, Chen W (2021). J Energy Chem.

[9] Black R, Oh S H, Lee J-H, Yim T, Adams B, Nazar L F (2012). J Am Chem Soc.

[10] Wong R A, Dutta A, Yang C, Yamanaka K, Ohta T, Nakao A, Waki K, Byon H R (2016). Chem Mater.

[11] Jung J-W, Cho S-H, Nam J S, Kim I-D (2020). Energy Storage Mater.

[12] Pham H T T, Choi Y, Park M-S, Lee J-W (2020). Chem Commun.

[13] Ottakam Thotiyl M M, Freunberger S A, Peng Z, Bruce P G (2013). J Am Chem Soc.

[14] Geng H, Peng Y, Qu L, Zhang H, Wu M (2020). Adv Energy Mater.

[15] Kichambare P, Kumar J, Rodrigues S, Kumar B (2011). J Power Sources.

[16] Shui J, Lin Y, Connell J W, Xu J, Fan X, Dai L (2016). ACS Energy Lett.

[17] Yang M, Zhou Y-N, Cao Y-N, Tong Z, Dong B, Chai Y-M (2020). Appl Mater Today.

[18] Wen X, Guan J (2019). Appl Mater Today.

[19] Liao Y-T, Nguyen V C, Ishiguro N, Young A P, Tsung C-K, Wu K C-W (2020). Appl Catal, B.

[20] Yang R-X, Bieh Y-T, Chen C H, Hsu C-Y, Kato Y, Yamamoto H, Tsung C-K, Wu K C-W (2021). ACS Sustainable Chem Eng.

[21] Konnerth H, Matsagar B M, Chen S S, Prechtl M H G, Shieh F-K, Wu K C-W (2020). Coord Chem Rev.

[22] Chueh C-C, Chen C-I, Su Y-A, Konnerth H, Gu Y-J, Kung C-W, Wu K C-W (2019). J Mater Chem A.

[23] Liu Y-C, Yeh L-H, Zheng M-J, Wu K C-W (2021). Sci Adv.

[24] Ge L, Yang Y, Wang L, Zhou W, De Marco R, Chen Z, Zou J, Zhu Z (2015). Carbon.

[25] Chen K, Sun Z, Fang R, Shi Y, Cheng H-M, Li F (2018). Adv Funct Mater.

[26] Sun X, Olivos-Suarez A I, Osadchii D, Romero M J V, Kapteijn F, Gascon J (2018). J Catal.

[27] Bhadra B N, Vinu A, Serre C, Jhung S H (2019). Mater Today.

[28] Chen Y-Z, Wang C, Wu Z-Y, Xiong Y, Xu Q, Yu S-H, Jiang H-L (2015). Adv Mater (Weinheim, Ger).

[29] Bhadra B N, Khan N A, Jhung S H (2019). J Mater Chem A.

[30] Qutaish H, Lee J, Hyeon Y, Han S A, Lee I-H, Heo Y-U, Whang D, Moon J, Park M-S, Kim J H (2021). Appl Surf Sci.

[31] Lee J, Park M-S, Kim J H (2021). Nano Convergence.

[32] Lee J, Choi S H, Qutaish H, Hyeon Y, Han S A, Heo Y-U, Whang D, Lee J-W, Moon J, Park M-S (2021). Energy Storage Mater.

[33] Hyeon Y, Lee J, Qutaish H, Han S A, Choi S H, Moon S W, Park M-S, Whang D, Kim J H (2020). Energy Storage Mater.

[34] Choi S H, Hyeon Y, Shin H R, Eom G H, Pham H T T, Whang D, Kim S Y, Lee J-W, Kim J H, Park M-S (2021). Nano Energy.

[35] Han S A, Lee J, Shim K, Lin J, Shahabuddin M, Lee J-W, Kim S-W, Park M-S, Kim J H (2018). Bull Chem Soc Jpn.

[36] Wang Y, Wang J, Mohamed Z, Huang Q, Chen T, Hou Y, Dang F, Zhang W, Wang H (2020). Appl Mater Today.

[37] Cao D-Q, Wang Q-Z, Yin X, Sun Y-D, Ma M, Wu Y-P, Liu X-J (2020). Energy Fuels.

[38] Zhao Y, Ding L, Wang X, Yang X, He J, Yang B, Wang B, Zhang D, Li Z (2021). J Alloys Compd.

[39] Saliba D, Ammar M, Rammal M, Al-Ghoul M, Hmadeh M (2018). J Am Chem Soc.

[40] Jung K-N, Kim J, Yamauchi Y, Park M-S, Lee J-W, Kim J H (2016). J Mater Chem A.

[41] Pham H T T, Kim Y, Kim Y-J, Lee J-W, Park M-S (2019). Adv Funct Mater.

