# Supplementary material for: Nanoarchitectonics of the cathode to improve the reversibility of Li–O2 batteries
Source: Beilstein J Nanotechnol. 2022 Jul 21;13:689–98. doi: 10.3762/bjnano.13.61 (PMC9344542; doi:10.3762/bjnano.13.61)
Supplement: File 1 — Additional TEM, EDS, XRD, XPS, BET, SEM, and cathodic overpotential measurements. [file Beilstein_J_Nanotechnol-13-689-s001.pdf]

BEILSTEIN JOURNAL OF NANOTECHNOLOGY

Supporting Information

for

Nanoarchitectonics of the cathode to improve the reversibility of Li–O_2 batteries

Hien Thi Thu Pham, Jonghyeok Yun, So Yeun Kim, Sang A Han, Jung Ho Kim, Jong-Won Lee and Min-Sik Park

Beilstein J. Nanotechnol. **2022,** *13,* 689–698. doi:10.3762/bjnano.13.61

Additional TEM, EDS, XRD, XPS, BET, SEM, and cathodic overpotential measurements

License and Terms: This is a supporting information file under the terms of the Creative Commons Attribution License (https://creativecommons.org/licenses/by/4.0). Please note that the reuse, redistribution and reproduction in particular requires that the author(s) and source are credited and that individual graphics may be subject to special legal provisions.
The license is subject to the *Beilstein Journal of Nanotechnology* terms and conditions: (https://www.beilstein-journals.org/bjnano/terms)

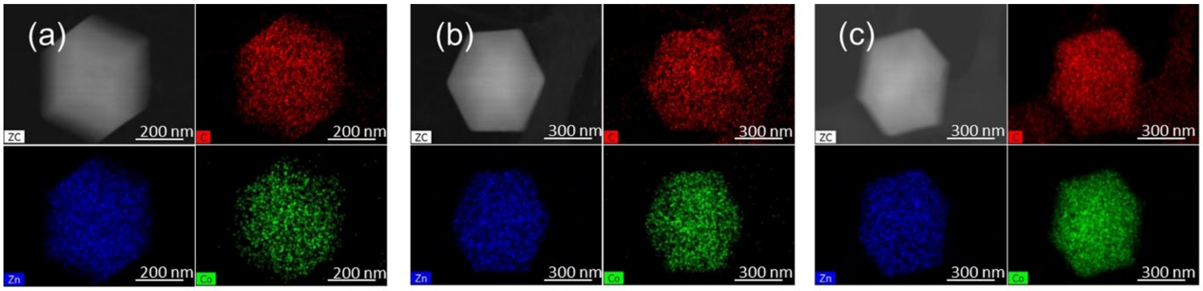

Figure S1: EDS elemental mapping results for (a) Zn_4Co_1/CNT, (b) Zn_1Co_1/CNT, and (c) Zn_1Co_4/CNT.

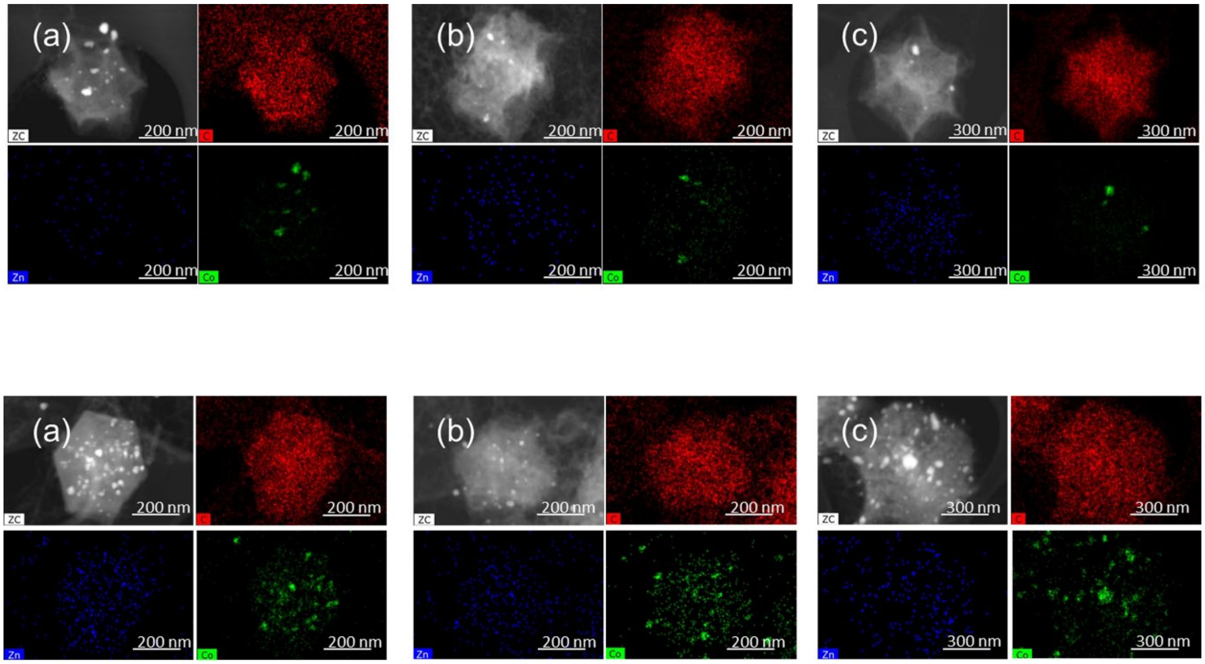

Figure S2: EDS elemental mapping results for (a) Zn_4Co_1–C/CNT, (b) Zn_1Co_1–C/CNT, and (c) Zn_1Co_4–C/CNT.

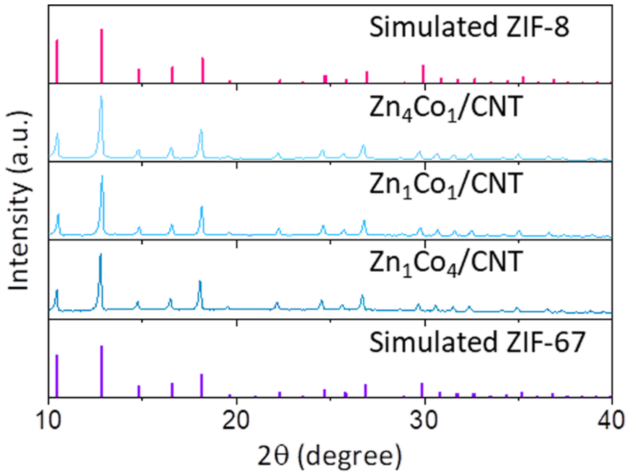

Figure S3: XRD patterns of as-synthesized Zn_xCo_y/CNT composites.

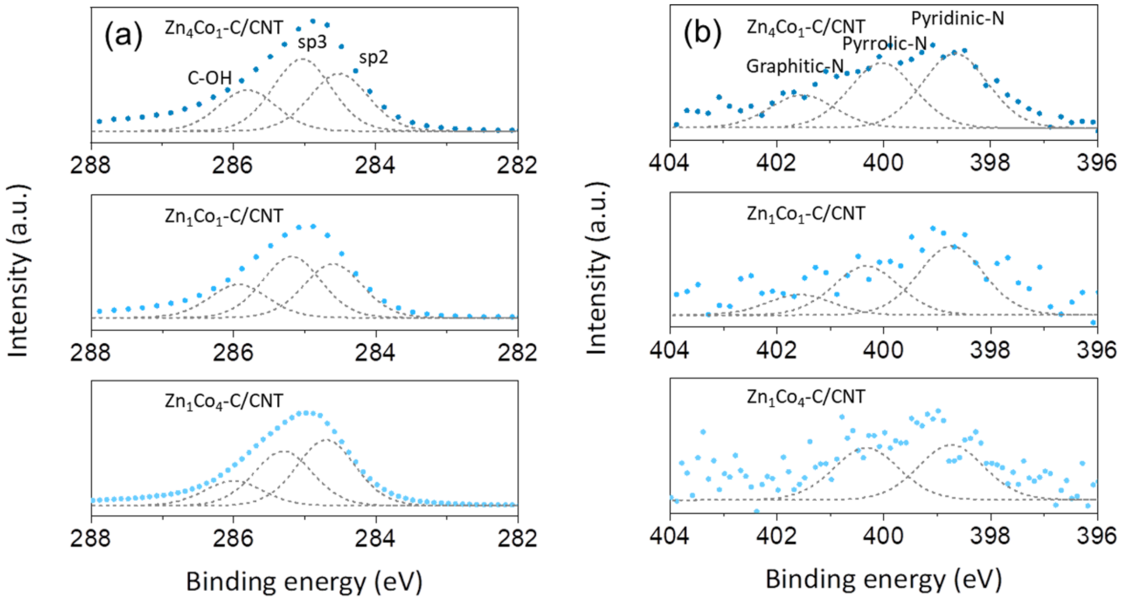

Figure S4: XPS spectra of Zn_xCo_y–C/CNT: (a) C 1s, and (b) N 1s.

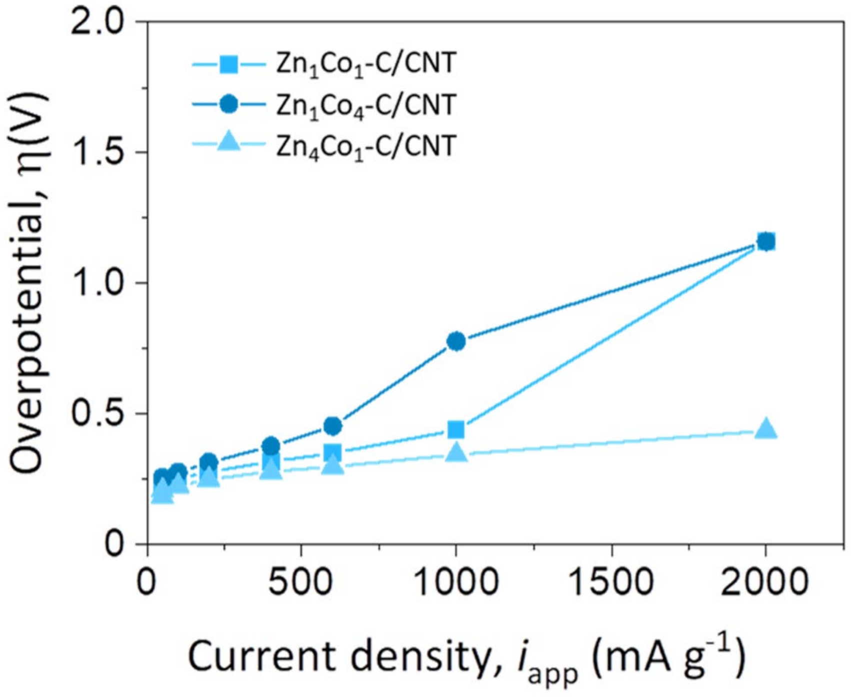

Figure S5: Cathodic overpotential (η) as a function of current density for LOBs with Zn_xCo_y–C/CNT electrodes. The cells were discharged in pure O_2 with a limited capacity of 500 mAh·g^{-1} at various current densities in the range of 50–2,000 mA·g^{-1}.

Table S1: Specific surface area and micro/meso/macropore volumes.

Material	BET surface area ($m^2 \cdot g^{-1}$)	Micropore volume ($cm^3 \cdot g^{-1}$)	Mesopore volume ($cm^3 \cdot g^{-1}$)	Mesopore volume ($cm^3 \cdot g^{-1}$)
Zn_1Co_4–C/CNT	305	0.04	1.23	0.05
Zn_1Co_1–C/CNT	357	0.03	0.95	0.05
Zn_4Co_1–C/CNT	489	0.07	0.88	0.04

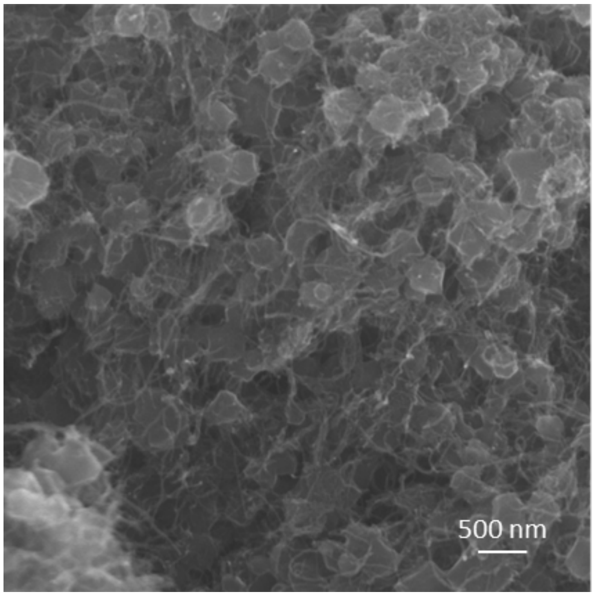

Figure S6: SEM image of the Zn_4Co_1–C/CNT electrode (charged) after 100 cycles.

Table S2: Electrochemical performance of cathodes based on CNTs, MOFs, or mesoporous carbon for Li-O_2 batteries.

Material	Current density	Depth of discharge	Number of cycles	Reference
Multiwalled CNT paper	250 mA·g^{-1}	1000 mAh·g^{-1}	50	S1
Multiwalled CNT foam	372 mA·g^{-1}	510 mAh·g^{-1}	100	S2
Ordered mesoporous carbon	200 mA·g^{-1}	2000 mAh·g^{-1}	25	S3
CNT	0.05 mA·cm^{-2}	0.5 mAh·cm^{-2}	73	S4
Mn–MOF-74	250 mA·g^{-1}	1000 mAh·g^{-1}	30	S5
MOF–C/CNT	200 mA·g^{-1}	500 mAh·g^{-1}	137	This work

S1. Chen, Y.; Li, F.; Tang, D.-M.; Jian, Z.; Liu, C.; Golberg, D.; Yamada, A.; Zou, H. J. Mater. Chem. A **2013**, 1, 13076-13081.

S2. Carter, R.; Oakes, L.; Cohn, A. P.; Holzgrafe, J.; Zarick, H. F.; Chatterjee, S.; Bardhan, R.; Pint, C. L. J. Phys. Chem. C **2014**, 118, 20137-20151.

S3. Park, J. B.; Lee, J.; Yoon, C. S.; Sun, Y. K. ACS Appl. Mater. Interfaces **2013**, 5, 13426-13431.

S4. Nomura, A.; Ito, K.; Kubo, Y. Sci. Rep. **2017**, 7, 45596.

S5. Wu, D.; Guo, Z.; Yin, X.; Pang, Q.; Tu, B.; Zhang, L.; Wang, Y. G.; Li, Q. Adv. Mater. **2014**, 26, 3258-3262.
